# The Rapid Carbapenemase Detection Method (rCDM) for Rapid and Accurate Detection of Carbapenemase-Producing *Enterobacteriaceae* and *Pseudomonas aeruginosa*

**DOI:** 10.3389/fcimb.2019.00371

**Published:** 2019-11-06

**Authors:** Xiaopeng Jing, Xiaochun Min, Xing Zhang, Lin Gong, Tingting Wu, Ruiling Sun, Liujun Chen, Rong Liu, Ji Zeng

**Affiliations:** ^1^Department of Clinical Laboratory, Wuhan Fourth Hospital, Puai Hospital, Tongji Medical College, Huazhong University of Science and Technology, Wuhan, China; ^2^Department of Disinfection and Pest Control, Wuhan Centers for Disease Prevention and Control, Wuhan, China; ^3^Key Laboratory of Ministry of Education for Neurological Disorders, Department of Pathophysiology, School of Basic Medicine, Tongji Medical College, Huazhong University of Science and Technology, Wuhan, China

**Keywords:** rapid carbapenemase detection method, simplified carbapenem inactivation method, carbapenemase, detection, *Enterobacteriaceae*, *Pseudomonas aeruginosa*

## Abstract

This study aimed to design a new method for rapid and accurate detection of carbapenemase phenotypes based on the simplified carbapenem inactivation method (sCIM). We evaluated the sensitivity and specificity of the method, called the rapid carbapenemase detection method (rCDM), for the detection of carbapenemase-producing *Enterobacteriaceae* and *Pseudomonas aeruginosa*. A total of 257 *Enterobacteriaceae*, 236 *P. aeruginosa*, and 20 *Acinetobacter baumannii* isolates were tested. Phenotypic evaluations were performed using rCDM, sCIM, and mCIM. For *Enterobacteriaceae*, the sensitivity of rCDM was 100% and the specificity was 99.6%. For *P*. *aeruginosa*, the sensitivity of rCDM was 97.4% and the specificity was 100%. Carbapenemase-producing *A. baumannii* were not detected by rCDM. The concordance rate of rCDM and sCIM for *Enterobacteriaceae* and *P. aeruginosa* was 99.8%, with the exception of one *P. aeruginosa* isolate that expressed the *bla*_*VIM*−4_ gene. The concordance rate of rCDM and mCIM for *Enterobacteriaceae* and *P. aeruginosa* was 100%. rCDM can be used to accurately detect carbapenemase-producing *Enterobacteriaceae* and *P. aeruginosa* in 5–6 h and is suitable for routine use in most clinical microbiology laboratories.

## Introduction

Carbapenemase-producing Gram negative bacilli are a major problem worldwide (Gupta et al., 2011). These bacteria cause high mortality and are associated with high treatment costs, often requiring a combination of multiple antibiotics for treatment (Centers for Disease Control Prevention, 2009; Magiorakos et al., 2017; Coope et al., 2018). Increased ability to detect carbapenemase-producing strains would greatly improve the prevention, control, and clinical treatment of nosocomial infections. Beginning in 2010, CLSI has proposed several testing methods, including the Carba NP, modified carbapenem inactivation method (mCIM), and EDTA-carbapenem inactivation method (eCIM) (CLSI, 2010, 2012b, 2017, 2018). The FDA also recommends methods such as matrix-assisted laser desorption ionization–time of flight mass spectrometry (MALDI-TOF MS) and other improved methods (Burckhardt and Zimmermann, 2011; Lee et al., 2013; Sun et al., 2017; Muntean et al., 2018). However, these procedures are lengthy (mCIM and eCIM require 24 h to detection), require expensive equipment such as MALDI-TOF MS, or involve reagents that cannot be stored for long periods of time (Carba NP, CarbaNP solution B can be stored in solution at 4–8°C for up to 3 days) (Burckhardt and Zimmermann, 2011; CLSI, 2018). In 2018, we improved on the mCIM method with the simplified carbapenem inactivation method (sCIM) (Jing et al., 2018). sCIM is easy to operate and the results are easy to observe; however, this method still takes 16–24 h to observe the results (Jing et al., 2018). In order to detect carbapenemase-producing strains in a shorter time, we have designed a new detection method, the rapid carbapenemase detection method (rCDM), that can detect carbapenemase-producing *Enterobacteriaceae* and *Pseudomonas aeruginosa* within 5–6 h based on antimicrobial disk susceptibility tests, and compared it with sCIM.

## Materials and Methods

### Bacteria

A total of 257 strains of *Enterobacteriaceae*, 236 strains of *P. aeruginosa*, and 20 strains of *Acinetobacter baumannii* were used to validate rCDM results. The strains were all clinical isolates derived from 10 different hospitals in China that were preserved in our laboratory. Of the 257 strains of *Enterobacteriaceae* resistant to cefotaxime, which included *Klebsiella pneumoniae* (52.9%), *Escherichia coli* (33.1%), *Enterobacter cloacae* (13.2%), and *Citrobacter freundii* (0.8%), 202 were resistant to imipenem, and 200 expressed the carbapenemase genes *bla*_*KPC*−2_ (56.5%), *bla*_*IMP*−4_ (19.0%), *bla*_*IMP*−2_ (1.5%), *bla*_*VIM*−1_ (5.5%), *bla*_*NDM*−1_ (16.5%), or *bla*_*OXA*−48_(1.0%) (Table 1). Of the 236 strains of *P. aeruginosa*, 196 were resistant to imipenem, and 38 expressed the carbapenemase genes *bla*_*KPC*−2_ (5.3%), *bla*_*IMP*−4_ (2.6%), *bla*_*VIM*−1_ (52.6%), *bla*_*VIM*−2_(7.9%), and *bla*_*VIM*−4_(31.6%). Twenty strains of *A. baumannii* were resistant to imipenem and produced OXA-23 carbapenemase (Table 2). All strains were identified at the species level using MALDI-TOF MS (Bruker Daltonics, Bremen, Germany) and a Vitek 2 compact instrument (bioMérieux, Hazelwood, MO, USA). Carbapenemase genes were detected in these strains using PCR and sequencing as previously described (Jing et al., 2018). Minimum inhibitory concentration (MIC) of imipenem was determined using broth microdilution method. *K. pneumoniae* ATCC 700603 was used as a quality control strain.

**Table 1:** Detection of carbapenemases in *Enterobacteriaceae* isolates by rCDM, mCIM, and sCIM.

***Enterobacteriaceae* (*n*)**	**PCR and sequencing (*n*)**	**MIC of imipenem (mg/L)**			**rCDM**		**sCIM**		**mCIM**	
		**≥4**	**2**	**≤1**	**+**	**–**	**+**	**–**	**+**	**–**
*K. pneumoniae* (136)	KPC-2 (79)	79	0	0	79	0	79	0	79	0
	IMP-4 (21)	21	0	0	21	0	21	0	21	0
	IMP-2 (1)	1	0	0	1	0	1	0	1	0
	VIM-1 (6)	6	0	0	6	0	6	0	6	0
	NDM-1 (7)	7	0	0	7	0	7	0	7	0
	OXA-48(1)	0	1	0	1	0	1	0	1	0
	None (21)	1	0	20	1	20	1	20	1	20
*E. coli* (85)	KPC-2 (33)	33	0	0	33	0	33	0	33	0
	IMP-4 (13)	13	0	0	13	0	13	0	13	0
	IMP-2 (2)	2	0	0	2	0	2	0	2	0
	NDM-1 (10)	10	0	0	10	0	10	0	10	0
	OXA-48 (1)	0	1	0	1	0	1	0	1	0
	None (26)	1	0	25	0	26	0	26	0	26
*E. cloacae* (34)	KPC-2 (1)	1	0	0	1	0	1	0	1	0
	IMP-4 (4)	4	0	0	4	0	4	0	4	0
	VIM-1 (5)	5	0	0	5	0	5	0	5	0
	NDM-1(14)	14	0	0	14	0	14	0	14	0
	None (10)	0	0	10	0	10	0	10	0	10
*C. freundii* (2)	NDM-1 (2)	2	0	0	2	0	2	0	2	0

*+, positive; –, negative; rCDM, rapid carbapenemase detection method; sCIM, simplified carbapenem inactivation method; mCIM, modified carbapenem inactivation method; MIC, minimum inhibitory concentration*.

**Table 2:** Detection of carbapenemase-producing *P. aeruginosa* and *A. baumannii* by rCDM, mCIM, and sCIM.

**Species (*n*)**	**PCR and sequencing (*n*)**	**MIC of imipenem (mg/L)**		**rCDM**		**sCIM**		**mCIM**	
		**≥8**	**≤2**	**+**	**–**	**+**	**–**	**+**	**–**
*P. aeruginosa* (236)	KPC-2 (2)	2	0	2	0	2	0	2	0
	IMP-4 (1)	1	0	1	0	1	0	1	0
	VIM-1 (20)	20	0	20	0	20	0	20	0
	VIM-2 (3)	3	0	3	0	3	0	3	0
	VIM-4 (12)	12	0	11	1	12	0	11	1
	None(198)	158	40	0	198	0	198	0	198
*A. baumannii* (20)	OXA-23(20)	20	0	0	20	20	0	0	20

*+, positive; –, negative; rCDM, rapid carbapenemase detection method; sCIM, simplified carbapenem inactivation method; mCIM, modified carbapenem inactivation method; MIC, minimum inhibitory concentration*.

### Modified Carbapenem Inactivation Method

mCIM was performed and interpreted as recommended by CLSI (2018).

### Simplified Carbapenem Inactivation Method

To perform sCIM for *Enterobacteriaceae*, a 0.5 McFarland standard suspension of *E. coli* ATCC 25922 was inoculated onto a Mueller Hinton agar (MHA) plate following the routine disk diffusion procedure. For *A. baumannii* and *P. aeruginosa*, a 0.5 McFarland standard suspension of *E. coli* ATCC 25922 was diluted 1:10 in saline and inoculated onto MHA plates following the routine disk diffusion procedure. Plates were allowed to dry for 3–10 min. Overnight colonies of test organisms were smeared onto an imipenem disk (10 μg; Oxoid, Hampshire, UK), with one side of the disk evenly coated with the test bacteria. The side of the disk containing bacteria was then placed onto the MHA plate previously inoculated with *E. coli* ATCC 25922. The plate was incubated for 16–18 h at 35°C in ambient air. A zone of inhibition with a diameter of 6–20 mm or satellite growth of *E. coli* ATCC 25922 colonies within a zone of inhibition ≤ 22 mm indicated that the isolate was capable of producing carbapenemase. A zone of inhibition ≥26 mm was considered a negative result. A zone of inhibition 23–25 mm was considered an inconclusive result (Jing et al., 2018).

### Preparation of Thin Mueller Hinton Agar (tMHA) Plates

To prepare tMHA plates, 38 g of MHA (Oxoid) was dissolved in 800 ml of distilled water. Distilled water was then added to a final volume of 1 L and the pH was adjusted to 7.4 ± 0.2. The solution was sterilized at 121°C for 15 min and poured into 90-mm sterile plates. Each plate was filled with 19 ml of MHA to a thickness of 3 mm. After cooling, the plates were stored at 4°C.

A 3.0 McFarland standard suspension of *E. coli* ATCC 25922 was inoculated onto a tMHA plate following the routine disk diffusion procedure. After settling for 5 min, imipenem disks (10 μg; Oxoid) were placed on the plates. The zone of inhibition was then measured by incubating at 35°C for 5 h in air. The diameter of the zone of inhibition of the 20 plates was measured for quality control.

### Rapid Carbapenemase Detection Method

**Figure 1:**
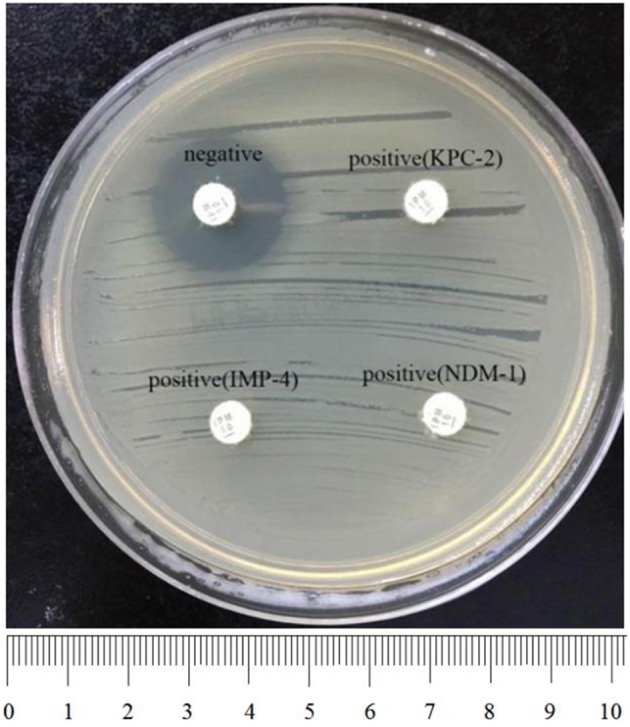
Results of rCDM testing of *Enterobacteriaceae* (6 h). The zone of inhibition of the negative isolate was 22 mm, whereas the zone of inhibition of the positive isolates was 6 mm. Upper right, KPC-2-producing *K. pneumoniae*; bottom left, IMP-4-producing *E. coli*; bottom right, NDM-1-producing *E. cloacae*.

**Figure 2:**
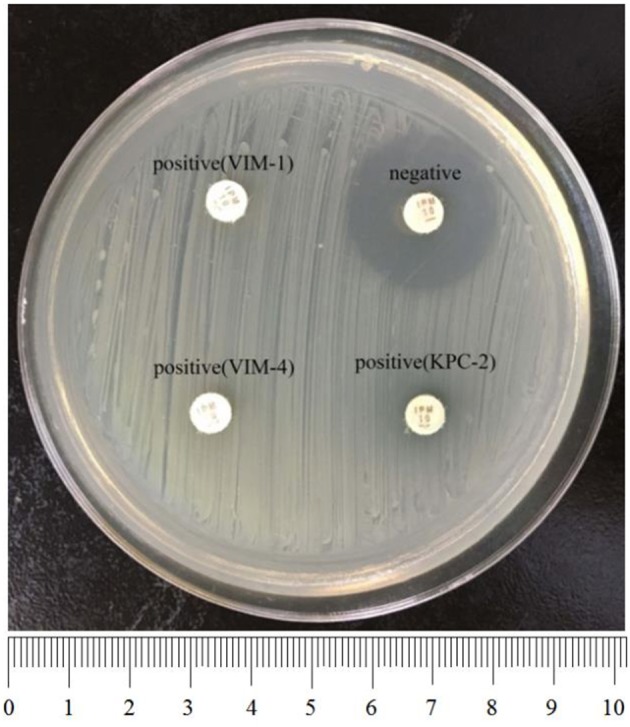
Results of rCDM testing of *P. aeruginosa* (6 h). The zone of inhibition of the negative isolate was 23 mm, whereas the zones of inhibition of the positive isolates were 6 mm. **Upper left**, VIM-1-producing *P. aeruginosa*; **bottom left**, VIM-4-producing *P. aeruginosa*; bottom right, KPC-2-producing *P. aeruginosa*.

**Figure 3:**
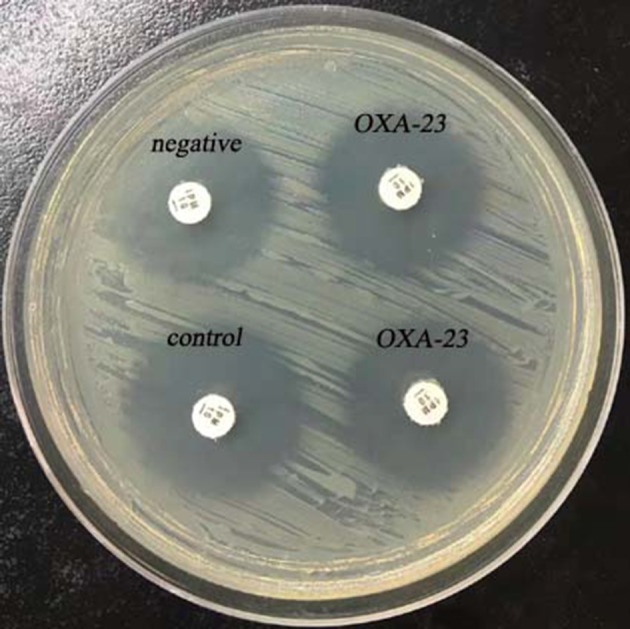
Results of rCDM testing of *A. baumannii* (6 h). The zone of inhibition of the control was 23 mm, and the zones of inhibition of the OXA-23-producing *A. baumannii* were 22 mm.

To perform rCDM, a 3.0 McFarland standard suspension of *E. coli* ATCC 25922 prepared using the direct colony suspension method was inoculated onto tMHA plates following the routine disk diffusion procedure. Plates were allowed to dry for 3–10 min. Then, one to three overnight colonies of test organisms grown on blood agar were smeared onto an imipenem disk (10 μg; Oxoid) to evenly coat one side of the disk with the test bacteria. Immediately afterwards, the side of the disk containing bacteria was placed on the tMHA plate previously inoculated with *E. coli* ATCC 25922. An imipenem disk without bacteria was placed on a tMHA plate as the control. All plates were incubated at 35°C for 5–6 h in ambient air. Bacterial strains producing carbapenemase can hydrolyze imipenem, allowing susceptible indicator strains to grow unchecked. A ≥5-mm decrease in a zone of inhibition for test organisms vs. control (Figures 1, 2) indicated that the isolate was capable of producing carbapenemase. A ≤ 3-mm decrease in a zone of inhibition for test organisms vs control (Figure 3) was considered to be a negative result; a 3–5 mm decrease in a zone of inhibition for test organisms vs. control was considered to be an indeterminate result.

### Statistic Analysis

McNemar test was used to examine the difference in the test results between rCDM and the three existing methods (PCR, sCIM, and mCIM), and the Kappa coefficients were further provided to indicate the degree of consistency. Data analysis was performed with SPSS 22.0. The significance was set at *p* < 0.05.

## Results

### Quality Control Range of Imipenem Disks on tMHA Plates

Disk diffusion quality control ranges for imipenem disks and *E. coli* ATCC 25922 on tMHA plates were between 22 and 24 mm. This range is only slightly smaller than the quality control range of the disk diffusion method using MHA plates.

### Sensitivity and Specificity of rCDM

Among the 257 tested strains of *Enterobacteriaceae*, 200 carbapenemase-producing *Enterobacteriaceae* were positive by rCDM, including KPC-2-producing (56.5%), IMP-4-producing (19.0%), NDM-1-producing (16.5%), VIM-1-producing (5.5%), IMP-2-producing (1.5%), and OXA-48-producing (1%) isolates. Of the 57 non-carbapenemase-producing *Enterobacteriaceae*, only one isolate was found to be positive by rCDM. The false positive strain was a *K. pneumoniae* isolate harboring CTX-M-15 (Table 1). The sensitivity of rCDM for *Enterobacteriaceae* was 100% and the specificity was 99.6%. The zones of inhibition for *Enterobacteriaceae* found to be negative for carbapenemase by rCDM ranged in size from 21 to 24 mm. In contrast, the zones of inhibition for *Enterobacteriaceae* found to be positive for carbapenemase by rCDM were 6 mm (Figure 1, Table 3). The zones of inhibition *for K. pneumoniae* ATCC 700603 assessed by rCDM were 21–22 mm.

**Table 3:** Comparison of rCDM, mCIM, and sCIM for selected strains.

**Strain (*n*)**	**Type of carbapenemase**	**rCDM zone diameter (mm)**	**sCIM zone diameter (mm)**	**mCIM zone diameter (mm)**
*Enterobacteriaceae* (200)	KPC-2, IMP-4, IMP-2, VIM-1, NDM-1, OXA-48	6	6	6
*P. aeruginosa* (35)	VIM-1, VIM-2, VIM-4, KPC-2, IMP-4	6	6	6–12
*P. aeruginosa* (2)	VIM-4	15	6	6
*P. aeruginosa* (1)	VIM-4	23	21^*^	22

*rCDM, rapid carbapenemase detection method; sCIM, simplified carbapenem inactivation method, mCIM, modified carbapenem inactivation method*.

* *Satellite growth observed around disk within the zone of inhibition*.

Among the 236 strains of *P. aeruginosa*, 37 of 38 carbapenemase-producing strains were positive by rCDM, including VIM-1-producing (52.6%), VIM-4-producing (31.6%), VIM-2-producing (7.9%), KPC-2-producing (5.3%), and IMP-4-producing (2.6%) isolates. Only one VIM-4-producing *P. aeruginosa* isolate with an MIC for imipenem of 8 mg/L was found to be negative by rCDM. The remaining 158 carbapenem-resistant strains and 40 carbapenem-sensitive strains were all negative by rCDM (Table 2). The sensitivity of rCDM for *P. aeruginosa* was 97.4% and the specificity was 100%. The zones of inhibition for *P. aeruginosa* found to be negative for carbapenemase by rCDM ranged in size from 21 to 24 mm. In contrast, the zones of inhibition for *P. aeruginosa* found to be positive for carbapenemase by rCDM were 6 mm, with the exception of two strains whose zones of inhibition were 15 mm (Figure 2, Table 3).

Twenty carbapenemase-producing *A. baumannii* isolates were found to be negative for carbapenemase by rCDM (Figure 3, Table 2) indicating that rCDM is not suitable for detection of carbapenemase-producing *A. baumannii*.

### Comparison of rCDM, mCIM, and sCIM

A total of 493 *Enterobacteriaceae* and *P. aeruginosa* strains were assessed by rCDM, mCIM, and sCIM. The concordance rate of rCDM and mCIM for *Enterobacteriaceae* and *P. aeruginosa* was 100% (Table 5). With the exception of one VIM-4-producing *P. aeruginosa*, the results of rCDM and sCIM were consistent for other bacteria. The concordance rate of rCDM and sCIM for *Enterobacteriaceae* and *P. aeruginosa* was 99.8% (Tables 1, 2, **5**, **7**). One *K. pneumoniae* isolate with an MIC for imipenem of 0.25 mg/L harbored CTX-M-15 and was found to be positive for carbapenemase by both rCDM and sCIM. One VIM-4-producing *P. aeruginosa* with an MIC for imipenem of 8 mg/L was found to be negative for carbapenemase by rCDM but positive for carbapenemase by sCIM. The zones of inhibition of this strain by sCIM were 21 mm with satellite growth. The 20 carbapenemase-producing *A. baumannii* were found to be negative for carbapenemase by rCDM and mCIM but positive by sCIM. Therefore, sCIM is superior to rCDM and mCIM for detection of carbapenemase-producing *A. baumannii*.

### Statistic Results

For *Enterobacteriaceae* and *P. aeruginosa*, there was no statistical difference between rCDM and the three existing methods (PCR, sCIM, and mCIM) (Tables 4–8). For *A. baumannii*, PCR/sCIM results and rCDM/mCIM results were statistically different (Table 9).

**Table 4:** The statistic results of sequencing and rCDM for 257 *Enterobacteriaceae* isolates.

**Sequencing**	**rCDM**		***P*_**McNemar**_**	**Kappa**
	**+**	**–**		
+	200	0	1	0.99
–	1	56		

*SPSS: χ^2^_McNemar_ = 0*.

**Table 5:** The statistic results of sCIM/mCIM and rCDM for 257 *Enterobacteriaceae* isolates.

**sCIM/mCIM**	**rCDM**		***P*_**McNemar**_**	**Kappa**
	**+**	**–**		
+	201	0	1	1.00
–	0	56		

*SPSS: χ^2^_McNemar_ = 0*.

**Table 6:** The statistic results of sequencing and rCDM for 236 *P. aeruginosa* isolates.

**Sequencing**	**rCDM**		***P*_**McNemar**_**	**Kappa**
	**+**	**–**		
+	37	1	1	0.98
–	0	198		

*SPSS: χ^2^_McNemar_ = 0*.

**Table 7:** The statistic results of sCIM and rCDM for 236 *P. aeruginosa* isolates.

**sCIM**	**rCDM**		***P*_**McNemar**_**	**Kappa**
	**+**	**–**		
+	37	1	1	0.98
–	0	198		

*SPSS: χ^2^_McNemar_ = 0*.

**Table 8:** The statistic results of mCIM and rCDM for 236 *P. aeruginosa* isolates.

**mCIM**	**rCDM**		***P*_**McNemar**_**	**Kappa**
	**+**	**–**		
+	37	0	1	1
–	0	199		

*SPSS: χ^2^_McNemar_ = 0*.

**Table 9:** The statistic results of sequencing/sCIM and rCDM/mCIM for 20 *A. baumannii* isolates.

**Sequencing/sCIM**	**rCDM/mCIM**		***P*_**McNemar**_**	**Kappa**
	**+**	**–**		
+	0	20	<0.001	0
–	0	0		

*SPSS: χ^2^_McNemar_ = 18.050*.

## Discussion

In the disk diffusion procedure, the growth of bacteria is inhibited at a certain concentration of antibiotic, and higher concentrations of the antibiotic are required to inhibit higher concentrations of bacteria. Because the thickness of tMHA plates (3 mm) is less than that of MHA plates (4 mm), the concentration of antibiotic in a disk differs between the two plates, and the concentration of antibiotics is higher on tMHA plates than on MHA plates at the same distance from the disk, which could result in inhibition of higher concentrations of bacteria (CLSI, 2012a). Therefore, when tMHA plates are used in the disk diffusion procedure, the concentration of test bacteria must be increased. Additionally, because higher concentrations of bacteria are inoculated, shorter incubation times are required for bacterial colonies to become visible. We designed rCDM based on these principles. In our experiments, we used tMHA plates and adjusted the concentration of *E. coli* ATCC 25922 to 3.0 McFarland standard. After 5–6 h, zones of inhibition were visible around the imipenem disk between 22 and 24 mm. These results confirmed that the imipenem disk could inhibit *E. coli* ATCC 25922 at a concentration of 3.0 McFarland standard and formed a larger zone of inhibition on tMHA plates.

When an imipenem disk is covered with carbapenemase-producing bacteria, imipenem is hydrolyzed and the diameter of the zone of inhibition is small. Because the Kcat values for imipenem are large for most carbapenemases, the rate of hydrolysis of imipenem is very fast, resulting in a significant decrease in the diameter of the zones of inhibition (Queenan and Bush, 2007). Refer to the extended-spectrum β-lactamase (ESBL) test criteria; in rCDM experiments, we considered a difference of ≥5 mm between the quality control zone of inhibition and the zone of inhibition of the positive strain as a positive result (CLSI, 2018).

In rCDM experiments, we found that the diameter of the zones of inhibition of carbapenemase-producing *Enterobacteriaceae*, including two OXA-48-producing strains, was 6 mm. Among the 38 carbapenemase-producing *P. aeruginosa*, 37 strains were carbapenemase-positive by rCDM and mCIM, with the exception of one VIM-4-producing *P. aeruginosa* strain. This strain was carbapenemase-positive by sCIM, but the diameter of the zone of inhibition was 21 mm and satellite growth was observed within the zone, indicating that this strain may produce only small amounts of carbapenemase. For carbapenemase-producing *A. baumannii*, rCDM and mCIM test results were negative. The OXA-23 type enzyme is the main carbapenemase produced by *A. baumannii*, and its ability to hydrolyze carbapenem is lower than that of other types of carbapenemases (Jean et al., 2015). Therefore, rCDM may not be suitable for the detection of strains with low carbapenemase activity. However, based on our experimental results, rCDM is suitable for the detection of carbapenemase-producing *Enterobacteriaceae* and *P. aeruginosa*.

Compared with other carbapenemase phenotype detection methods, rCDM has several distinct advantages. First, rCDM does not require special equipment or reagents. Second, rCDM is simple to perform. Third, the results are easy to assess. Finally, the detection time of rCDM is only 5–6 h, 1 day shorter than sCIM or mCIM. rCDM has a wide range of detection, and can be used to routinely detect carbapenemase-producing *Enterobacteriaceae* and *P. aeruginosa* in clinical microbiology laboratories if tMHA could achieve commercial supply.

## Data Availability Statement

All datasets generated for this study are included in the article/supplementary material.

## Author Contributions

XJ, XM, XZ, LG, TW, RS, and LC isolated bacteria and performed the laboratory measurements. JZ and XJ made substantial contributions to conception and design. JZ and RL wrote and revised the manuscript. JZ drafted the manuscript. All authors read and approved the final manuscript.

### Conflict of Interest

The authors declare that the research was conducted in the absence of any commercial or financial relationships that could be construed as a potential conflict of interest.
